# Extraction of polysaccharides from *Fomes officinalis* Ames and their antitumor activity

**DOI:** 10.3892/etm.2013.1163

**Published:** 2013-06-17

**Authors:** MINGDONG HU, HUIFENG ZHANG, BO FENG, KE LIU, SHUYING GUO

**Affiliations:** 1 Institute of Respiration, The Second Affiliated Hospital, The Third Military Medical University, Chongqing 400037;; 2 College of Life Sciences, Jilin University, Changchun, Jilin 130013;; 3 Pharmaceutical Analysis Department, Jilin Medical College, Jilin, Jilin 132013, P.R. China

**Keywords:** *Fomes officinalis* Ames, polysaccharide, extraction, S180 tumor-bearing mice, immune function

## Abstract

The aim of this study was to optimize the extraction parameters of *Fomes officinalis* Ames polysaccharides (FOAPs) and evaluate their antitumor activity. FOAPs were extracted using the hot water extraction, acid extraction and alkali extraction methods, respectively. Alcohol precipitation and acetone washes were conducted to separate and purify the FOAPs. The FOAP content was determined using the phenol-sulfuric acid method. The effects of raw material particle size, extraction time and material-liquid ratio on the yield of FOAPs were investigated, and the effects of FOAPs on the immune function of S180 tumor-bearing mice and their antitumor activity were evaluated. The yield of FOAPs obtained with the hot water extraction method was higher compared with the yields of the other methods. The optimum extraction conditions were as follows: a raw material particle size of 24 mesh; an extraction time of 2.5 h; and a material-liquid ratio of 1 g:12 ml. Under these conditions, the yield of FOAPs was 1.13%. FOAPs significantly inhibited tumor growth and enhanced the immune function in S180 tumor-bearing mice. FOAPs extracted using the hot water extraction method have antitumor activity.

## Introduction

Cancer is the second leading cause of mortality worldwide. The mortality rate of cancer is only inferior to that of cardiovascular disease (1). The threat of cancer to human health has not previously been effectively controlled. Chemotherapy is the main treatment for cancer, but its therapeutic effect is severely limited by side-effects and multidrug resistance (2). Traditional Chinese medicine has a long history of use in the treatment of diseases. ‘Shen Nong’s Herbal Classic’ and other herbal monographs have documented and discussed multiple traditional Chinese medicine prescriptions for treating severe ulcers and malignant tumors. Traditional Chinese medicine has notable advantages of multi-component, multi-link and multi-target for treating cancer (3). The extraction of active antitumor ingredients from Chinese herbal medicines has gained an increasing amount of attention.

In recent years, polysaccharides of plant origin have emerged as an important class of bioactive natural products. A wide range of polysaccharides have been reported to exhibit properties, including antioxidant activity (4,5), free radical-scavenging activity (6), the enhancement of immune capacity (7,8), the ability to lower blood sugar and blood lipids (9,10) and anti-viral activity (11). Furthermore, polysaccharides have been shown to exert antitumor activity by enhancing the immune capacity of the body and inducing tumor cell apoptosis (12–15). The majority of methods for extracting polysaccharides involve hot water extraction and low temperature alkali extraction (16,17). Research has revealed that plant polysaccharides extracted with hot water exhibit *in vivo* and *in vitro* antitumor activities (18). The extraction yields differ depending on the plants and extraction methods used.

*Fomes officinalis* Ames (‘Kubaiti’ in Chinese), is the dried fruiting body of the *Fomes officinalis* fungus, and is commonly used as a medicine by Uyghur doctors in China. It has many functions such as warming lung, eliminating phlegm, relieving asthma, activating blood and dispersing swelling, inducing diuresis, enhancing physical strength, prolonging the anti-fatigue and hypoxia tolerance time and improving the emergency response capacity of the body. It is often used for treating chronic bronchitis, abdominal pain, influenza, tuberculosis and cancer (19). As reported by Wu *et al* (20), terpene and steroid compounds have been identified to be the main active components of *Fomes officinalis* Ames, and are related to the observed efficacies. Polysaccharides from medicinal fungi and their derivatives have become important in immune regulation and cancer treatment. The *Fomes officinalis* Ames polysaccharides (FOAPs) have also obtained increasing attention (21,22). However, studies concerning the extraction of FOAPs and their efficacy have been seldom reported. In the current study, the extraction methods of FOAPs and the anti-tumor activities of the extracts were investigated. The objective was to provide a theoretical foundation and experimental basis for the study of other drugs derived from fungi.

## Materials and methods

### Apparatus and reagents

The main apparatus and reagents were as follows: 1700 UV-Vis spectrophotometer (Shimadzu, Kyoto, Japan), RE-3000 rotary evaporators (Shanghai Yarong Biochemical Instrument Factory, Shanghai, China), TD-1500 low speed centrifuge (Hunan Kaida Scientific Instruments Co., Ltd, Changsha, China), FA1104N electronic balance (Shanghai Precision & Scientific Instrument Co., Ltd, Shanghai, China), GSY-II thermostatic water bath (Beijing Medical Equipment Factory, Beijing, China) and *Fomes officinalis* Ames (Ningbo Dekang Biological Products Co., Ltd, Ningbo, China). Other reagents were analytically pure, and deionized water was used in all experiments.

### Animals

Clean Kunming mice inoculated with S180 tumor cells were provided by the Key Laboratory of Forest Plant Ecology of Ministry of Education, Northeast Forestry University (Heilongjiang, China). The study was approved by the ethics committee if The Second Hispital Affiliated to The Third Military Medical University of PLA (Chongqing, China).

### Identification of Fomes officinalis Ames

*Fomes officinalis* Ames was identified by the following methods: a) 0.1 g *Fomes officinalis* Ames was placed in a bottle and 2 ml ethyl acetate was added. Following agitation and impregnation for 1 h, the mixture was filtered. The fluorescence of the filtrate was observed with a spectrophotometer. b) The filtrate (0.5 ml) was placed into a test tube, and 0.5 ml sulfuric acid was added slowly along the tube wall. The color of the mixture was observed, and then the fluorescence of the mixture was observed with the spectrophotometer.

### Extraction of FOAPs

Raw *Fomes officinalis* Ames was pulverized and the powder was dried at 80°C for 2 h. Dried powder (25 g) was placed in a flask (500 ml), followed by 125 ml deionized water. The mixture was refluxed for 2 h and subsequently filtered. The extraction was repeated twice, and the three filtrates were mixed. Chloroform was added to the mixed filtrate in order to remove proteins and pigments. After discarding the chloroform layer, a 95% ethanol solution was added to induce precipitation. Following centrifugation (1227 × g, 8 min), washing with acetone and drying, the FOAPs were obtained.

For the acid and alkali extraction methods, the extractions were performed at room temperature, and hydrochloric acid solution and sodium hydroxide solution were used as the respective extraction solvents. Following extraction, the extraction solution was immediately neutralized. Other procedures were the same as those used in the hot water extraction method.

### Determination of FOAP yield

A standard glucose solution (250 ml, 0.2 mg/ml) was prepared and 2.5, 5, 7.5, 10, 12.5, 15 and 17.5 ml of the standard solution were diluted to 50 ml with distilled water, respectively. Subsequently, 2 ml diluted solution was added to a test tube. Then, 1 ml 6% phenol reagent was added and the mixture was fully blended, and 5 ml concentrated sulfuric acid was added quickly. After standing for 5 min, the mixture was incubated in a boiling water bath for 15 min, and then cooled down to room temperature. Concurrently, a reagent blank control experiment was performed. The absorbance of the mixture was determined at 490 nm using a UV-Vis spectrophotometer. The standard glucose curve was established by plotting the absorbance value (y-axis) against the glucose concentration (x-axis, mg/ml). The regression equation took the form: y=16.314× − 0.0619, r=0.9992.

The yield of FOAPs was expressed as follows: Yield (%) = (m/M) × 100 (1), where m is the weight of FOAPs analyzed by UV-Vis analysis (g), and M is the weight of *Fomes officinalis* Ames (g).

### Weighing of the organs of the immune system and determination of the tumor inhibition rate

A cell suspension (1×10^6^/ml) of S180 mouse ascites tumor cells (seven days following vaccination) was prepared with saline. Subsequently, 0.2 ml cell suspension was subcutaneously inoculated into the right axillary region of the mice. The mice were randomly divided 24 h later into the normal group, the model group, the fluorouracil (5-Fu) group and three FOAP groups, with 10 mice per group. The three FOAPs groups were intragastricly administrated with FOAPs at doses of 50, 100 and 200 mg/(kg·d), respectively. Equal volumes of saline were administered to the normal and model groups and the 5-Fu group was treated with 5-Fu (30 mg/kg·d). All irrigations were conducted for seven consecutive days. On the eighth day, all mice were sacrificed. The tumor, thymus and spleen were dissected and weighed. The tumor inhibition rate, thymus index and spleen index were determined according to previously described methods (23), as follows: Tumor inhibition rate (%) = [1 - (mean tumor weight of treatment group/mean tumor weight of model group)] × 100%. Thymus index (mg/g) = (thymus weight/body weight) × 1,000. Spleen index (mg/g) = (spleen weight/body weight) × 1,000.

### Statistical analysis

Data are expressed as the mean ± SD. Statistical analyses were performed using SPSS 13.0 statistical software (SPSS, Inc., Chicago, IL, USA). A t-test was used to analyze the differences between two groups. P<0.05 was considered to indicate a statistically significant difference.

## Results and Discussion

### Identification of Fomes officinalis Ames

According to the identification method a), the filtrate displayed light blue fluorescence under a UV light. In method b), following the addition of sulfuric acid, the filtrate exhibited green fluorescence and yellow fluorescence was observed under UV light (365 nm). The *Fomes officinalis* Ames material met the criteria specified in Standard of Medicine PRC-Uygur Medicine Fascicule (24).

### Effects of various extraction methods on the yield of FOAPs

**Figure 1.**
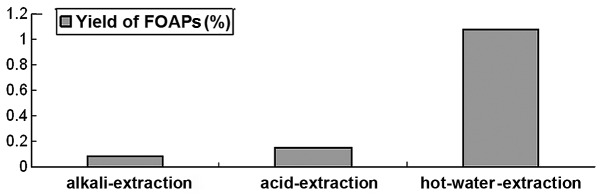
Effects of different extraction methods on the yield of *Fomes officinalis* Ames polysaccharides (FOAPs).

The effects of various extraction methods on the yield of FOAPs are shown in Fig. 1. The yield of FOAPs obtained by the hot water extraction method was higher than that by the acid and alkali extraction methods. Water is a safe and economic extraction solvent. It effectively penetrates plant tissue, resulting in a high extraction yield. In the acid or alkali extraction method, the acid or alkali may cause glycoside bonds in the polysaccharide to rupture, resulting in a low yield. Therefore, following acid or alkali extraction, the extraction solution should be neutralized immediately, concentrated and precipitated in order to maximize the yield of polysaccharides. In this study, the hot water extraction method was suitable for extracting FOAPs. There are many methods for the extraction and separation of polysaccharides. The optimum extraction method and technology should be determined according to the characteristics of the polysaccharide, its physical and chemical properties and its experimental results.

### Effects of raw material particle size on the yield of FOAPs

**Figure 2.**
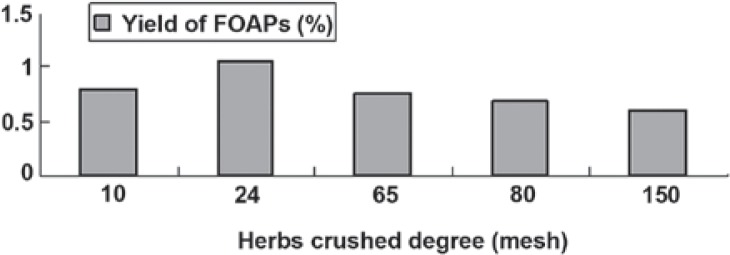
Effects of raw material particle size on the yield of *Fomes officinalis* Ames polysaccharides (FOAPs).

The effect of the particle size distribution of the raw material (10, 24, 65, 80 and 150 mesh) on the FOAP yield was investigated using an extraction time of 2 h and material-liquid ratio of 1 g:10 ml. The results are shown in Fig. 2. In general, as the particle size increased, the FOAP yield decreased. The yield was at its highest (1.05%), however, when the particle size was 24 mesh rather than 10 mesh. The reason for this may be that too small a particle size enhances the interaction between the material particles, resulting in a low yield. Furthermore, a too small particle size may increase the difficulty of follow-up filtration. Therefore, 24 mesh was selected as an optimum raw material particle size for subsequent experiments.

### Effects of various extraction times on the yield of FOAPs

**Figure 3.**
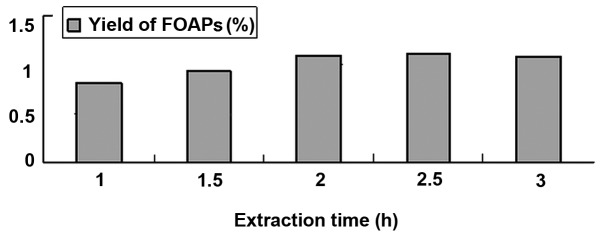
Effects of different extraction times on the yield of *Fomes officinalis* Ames polysaccharides (FOAPs).

Extraction times ranging from 1 to 3 h were investigated, with a 24 mesh raw material particle size and a material-liquid ratio of 1 g:10 ml. The results are shown in Fig. 3. The yield of FOAPs increased with increasing extraction time, but began to reduce when the extraction time was >2.5 h. This indicates that when the extraction time is too long, greater amounts of other components are extracted, resulting in a lower FOAP content. Furthermore, too long an extraction time may increase energy consumption. Considering the feasibility in a practical application, 2.5 h was selected as a suitable extraction time.

### Effects of various material-liquid ratios on the yield of FOAPs

**Figure 4.**
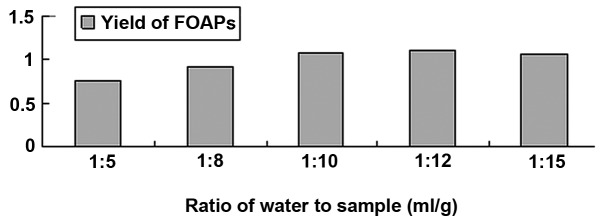
Effects of different material-liquid ratios on the yield of *Fomes officinalis* Ames polysaccharides (FOAPs).

The effect of various material-liquid ratios (g:ml; 1:5, 1:8, 1:10, 1:12 and 1:15, respectively) on the yield of FOAPs was investigated (extraction time was 2 h and raw material particle size was 24 mesh). As shown in Fig. 4, the yield of FOAPs was at its highest when the material-liquid ratio was 1 g:12 ml. There should be sufficient liquid to fully dissolve the polysaccharide, but the amount of liquid should be as small as possible, in order to reduce the time taken and the energy consumption during concentration. Based on these results, 1 g:12 ml was selected as a suitable material-liquid ratio.

### Verification experiment

Triplicate experiments were performed under the optimum hot water extraction conditions: a 24-mesh raw material particle size, a 2.5-h extraction time and a material-liquid ratio of 1 g:12 ml. The average yield of FOAPs was 1.13%.

### Effects of FOAPs on immune function and their antitumor activity in mice

**Figure 5.**
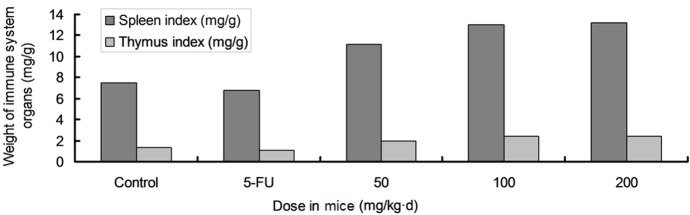
Effects of different doses of *Fomes officinalis* Ames polysaccharides (FOAPs) on the weight of immune system organs in mice. 5-Fu, 5-fluorouracil.

**Figure 6.**
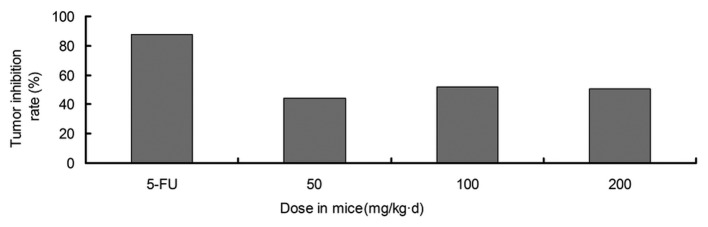
Effects of different doses of *Fomes officinalis* Ames polysaccharides (FOAPs) on the tumor inhibition rate in mice. 5-Fu, 5-fluorouracil.

Effects of various doses of FOAPs on the weight of the organs of the immune system and the tumor inhibition rate in S180 tumor-bearing mice are shown in Figs. 5 and 6, respectively. FOAPs did not affect the survival and body weight of the S180 tumor-bearing mice, but inhibited tumor growth. There was a significant difference in the tumor weight between each FOAP group and the model group (P<0.05). Treatment with FOAPs increased the thymus and spleen weights of the S180 tumor-bearing mice. Compared with the 5-Fu group, the tumor inhibition rate in the FOAPs groups was lower, but there was a more marked stimulation of the organs of the immune system. Though 5-Fu effectively inhibited tumor growth, it markedly suppressed the immune system. The combination of FOAPs and 5-Fu may result in enhanced antitumor effects and simultaneously reduce the poisonous side-effects of 5-Fu.

In conclusion, the hot water extraction method is suitable for extracting FOAPs. The optimum extraction conditions were as follows: a 24-mesh raw material particle size, a 2.5-h extraction time and a 1 g:12 ml material-liquid ratio. Chloroform removed the proteins and pigments from the extraction solution. Under these conditions, the yield of FOAP was 1.13%. FOAPs inhibit tumor growth and enhance the immune function in mice.
